# Primary care reform and concurrent changes in the regional distribution and equity of the ministry of health family medicine workforce in Saudi Arabia: a descriptive longitudinal analysis, 2014–2022

**DOI:** 10.3389/frhs.2026.1962491

**Published:** 2026-10-13

**Authors:** Mohammed S. Alyousef

**Affiliations:** Department of Health Services Administration and Hospitals, Faculty of Economic and Administration, King Abdulaziz University, Jeddah, Saudi Arabia

**Keywords:** family medicine, health equity, health reform, health services, health workforce, primary care, primary care reform, Saudi Arabia

## Abstract

**Background:**

Saudi Arabia's Vision 2030, with family medicine positioned as the organising specialty of primary care. Whether this transformation has translated into a more equitable distribution of family medicine across the Kingdom remains under-examined. This study aimed to evaluate whether the primary-care reform altered the regional distribution and equity of the FP workforce across the fourteen health administrative units of the Kingdom.

**Methods:**

A retrospective, longitudinal panel was constructed from Saudi Ministry of Health Statistical Yearbooks (2014–2022) across three reform phases: pre-reform (2014–2016), transformation (2017–2020), and post-reform (2021–2022). FM density per 10,000 population was the primary outcome. Inequality was assessed by the Gini coefficient, Theil index, and coefficient of variation, (bootstrap 95% Cis: 1,000 resamples). Phase comparisons used by Kruskal–Wallis with Bonferroni-adjusted Mann–Whitney pairwise tests; temporal trends by Mann–Kendall. Regional trajectories by Ward hierarchical clustering on z-standardised density paths. Because an administrative reclassification in 2021 depressed the national count, trend results are reported both for the full series and with 2021 excluded, and the artifact-adjusted estimates are treated as the primary ones. Only MOH-employed family physicians are covered; private, military and university providers are outside the data source.

**Results:**

National FP density rose from 0.96 to 1.47 per 10,000 (+53.0%), with absolute headcount increasing from 2,625 to 4,273 (+62.8%). Mean regional density grew faster than national density (+82.4%), indicating proportionally larger gains in less populous regions. All three inequality measures were higher in 2022 than in 2014: Gini +31.5%, Theil +39.1%, and coefficient of variation +28.6%. The Mann–Kendall trend for the Gini coefficient and the coefficient of variation was only marginally significant (*p* = 0.048), did not hold once 2021 was excluded (*p* = 0.108), and the Theil trend was not significant (*p* = 0.118); the widening is therefore suggestive rather than statistically robust. Phase-level mean densities differed significantly across phases (Kruskal–Wallis *H* = 15.21, *p* < 0.001), with each successive phase associated with higher density and a more unequal distribution. Hierarchical clustering identified three trajectory groups: a cluster of large urban units with stagnant or declining MOH density (Jeddah, Makkah, Al-Ahsa, Hail, Hafar Al-Batin), a strong-growth peripheral cluster (Bisha +262.1%, Northern Borders +122.9%, Madinah, Tabuk, Qassim, Taif, Asir, Eastern), and Riyadh as a singleton with a peak-and-decline trajectory.

**Conclusions:**

The health reform period was accompanied by substantial absolute growth in the MOH family medicine workforce and by an increase in regional inequality between 2014 and 2022 that is suggestive but not statistically robust. Within the MOH workforce, gains concentrated in peripheral units while the major urban corridors stagnated, a pattern that differs from the urban bias reported in most international settings; because non-MOH providers are concentrated in those same cities and are not captured here, this should not be read as a reversal of urban bias in total family physician supply. Translating Vision 2030's quantitative gains into the equitable workforce envisioned under “a family physician for every family” will require region-specific retention and deployment strategies rather than further aggregate growth alone.

## 1 Introduction

Family medicine is the backbone of high-quality health systems and is of particular interest to the World Health Organization. The WHO describes a robust health system as a health workforce that is adequately sized and equitably distributed to provide the necessary health services (1). Increased investment in primary health care is associated with an improvement in population health and a positive return for health systems (2). Enhanced primary health care is associated with better health outcomes in various health indicators in several European countries (3). Family medicine is central to the health systems and delivers person-centered, continuous, and comprehensive care. A consensus of sorts exists globally, but most countries have inadequate family medicine specialization workforce with an inequitable and negative distribution in terms of health systems access, care, quality, and health inequities (4).

Over the last decade, the ambitious health reform initiated by Saudi Arabia has positioned the country to undertake one of the largest health reforms in the region. The new Model of Care in Saudi Arabia, with the broader Vision 2030, places Family Medicine as a priority. The Vision 2030 agenda prioritizes the development of health infrastructure, an increase in the health workforce, a strategic re-orientation of services to address health needs, and the diversification of services, all of which are synonymous with family medicine (5–9). On the national level, this transformation has been characterized by the tangible growth of new health facilities, new personnel, increased rural coverage, and a widened screening focus on chronic diseases (6). There are still concerns on whether this transformation has been equitably distributed across the Kingdom.

In the last decade, as a result of the health reforms undertaken by Saudi Arabia, the Kingdom is now poised to execute one of the largest health reforms in the region. The New Model of Care in Saudi Arabia, aligned to Vision 2030, places Family Medicine as a priority. The Vision 2030 agenda seeks to grow health infrastructure and create a flexible health workforce and to orientate and diversify health services—all of which fit the bill of family medicine (5–9). At the national level, this has generally been reflected in new health facilities, new personnel, better health care access in rural areas, and a focus in chronic disease screening (6). However, it is still unclear if this transformation has been equitably achieved in the whole Kingdom.

### 1.1 Primary care in Saudi arabia before the family medicine model

To fully understand the factors that led to the change, one must know the context in which these factors arose. Up until the 2000–2000s, the Ministry of Health was providing primary care by managing around two thousand primary healthcare centers. The approx twenty, regional health directorates, were under direct ministry supervision and were linked by budgets with historically fixed allocations (10, 11). Care at primary health care centers remain free to all Saudi citizens and public sector employees and financed from the general budget rather than from insurance or other specific health taxes (10, 11). Staffing was done within the health facility rather than serving the population; a health center was constructed and a post was linked to it.

The post was filled by the first available physician, who was typically an expatriate general practitioner (12, 13). Family physicians were estimated to make up one in ten of the primary care medical workforce at the end of the period studied by Al-Khaldi and colleagues (14). There was neither patient registration with an assigned physician nor a practice catchment population, and visiting patients skipped primary care by going directly to a hospital clinic. Patients who were dissatisfied with a center went directly to hospital outpatient departments and emergency departments (6, 10).

Continuity was a matter of habit as opposed to a matter of structure. The context is important here: the absence of empanelment and a primary care gate, and the presence of a facility-based, generalist-staffed primary care tier. It is against this that the shift toward a family medicine model, and the later consolidation of directorates into regional health clusters, must be appreciated. Only when the specialty is expected to take first-contact responsibility for a defined population does the question of even distribution of family physicians become relevant, which is, of course, the aim of the health transformation (7, 15).

Data on workforce distribution is vague. At the national level, it shows a persistent problem of structural workforce shortages and distribution gaps (10, 12, 13, 16, 17). In regard to distribution of centers vs. distribution of physicians, a consistent finding of research has been an uneven regional distribution, in which some peripheral areas had better, worse, and poorer facility-to-population ratios, and poor and imbalanced distribution and palliative care services (5, 18–22). At the level of physician workforce, Kattan and Alshahrani (23) illustrated regional disproportion in the total physician workforce for 2022. Meanwhile, Qattan and Kattan (24) reported a similar distribution among obstetrician-gynecologists. Neither study examined family medicine, and none gave a long-term account covering the whole period of the reform.

The administrative reorganization that accompanied the clinical reform warrants independent theory development because it makes decisions about workforce allocation. Bossert's decision-space approach defines decentralization as the range of control in financing, service organization, human resources, access, and governance. The range of discretion can be from complete to no control. The value of the approach for the workforce lies in distinguishing authority from the capacity to use it. A cluster may be endowed with broad discretion for human resource control. Authority have been given to control managing human resources (25–27). Kolehmainen-Aitken's analysis of the health workforce from the personnel perspective comes to a similar conclusion. She states that the transfer of authority for human resource management occurs more quickly than the actual transfer of control, and staffing equity is one of the first casualties when the two fall out of synch (28). The health clusters created in Saudi Arabia from 2021 onward should be seen as an early and partial devolution of this kind. Clinical and operational responsibility has been assigned to the clusters, while the terms and conditions of employment of physicians has not followed (11). The framework would expect that in such conditions, clusters will progress at different rates, and a period of divergence would be expected before convergence, if convergence were to occur. It is Distribution that forms the basis of equity and trust in Primary Care.

This section defines the term inequality as used in this paper, since the term has multiple connotations in health policy. In this context, Whitehead's differentiation remains the most useful (49). Not all variances in health or health related resources are inequitable. For policy, the focus is on the avoidable and unnecessary types of inequity and inequities that are also unfair. The inequality of interest in this paper is even more constrained. This inequality in supply refers to the number of family physicians available relative to the resident population in each administrative area, and thus speaks to the dimension of accessibility; it has no bearing on accessibility, acceptability, affordability, or use. In this context, price is not the barrier to care at Ministry of Health facilities, which are free at the point of use. The barrier, and thus the constraint, to accessing a family physician, in this case, is geographical and organizational. The constraint is whether a physician is located in the area at all and how far the patient must travel. The unit of interest is the administrative area not the individual. Hence, the results do not address intra-area disparity. One region may have an adequate ratio of family physicians while rural areas in that region may remain underserved. The criteria used by the Ministry to site new facilities and post new staff, including population size and density, coverage of existing facilities, road accessibility, and regional demand, are of even finer scale than the scale considered in this paper. This is discussed in the Discussion section.

This study attempts to fill this void. Most equity-related analyses have examined the placement of PHC centres, the total supply of physicians, and the specialty workforce distribution, and have mainly used short-term and/or cross-sectional data (5, 13, 18, 20–24). A national, longitudinal study of family medicine encompassing the entire post-2014 reform period is absent. Given the clear role of family medicine in the new Model of Care, an analysis of this nature is directly pertinent to workforce planning. This study aims to provide evidence for the development of differentiated regional policies of family medicine and to implement the “a family physician for every family” initiative. It also provides early-stage evidence on the equity component of the Saudi primary care reform. This study examines national administrative data (2014–2022) provides the first analysis to describe the density and regional distribution of MOH-employed family physicians in Saudi Arabia. The design is descriptive; it documents changes that occurred alongside the reform and does not estimate what would have happened in its absence. To measure family physician distribution in the fourteen regional health administrative sections of the Ministry of Health, we use the Gini coefficient and Theil index (29). We examined qualified family physicians in primary health care across the three reform phases: pre-reform (2014–2016), transformation (2017–2020), and post-reform (2021–2022).

Two factors comprise the interest of the question. First, consider arithmetic of policy. Committing to assign a family physician to each family is a commitment on people, not on numbers. The split between the most and least served regions widens. The two things can happen concurrently; only a measurement of distribution can separate them. The second factor has to do with information. The information that is available on the Saudi primary care workforce is from counts of facilities, from the physician workforce as one single block, or from snapshots taken of this workforce after the system of care was reformed. None of these methods can determine the impact of the reform since none of them studied the system prior to the reform. Family medicine, the specialty that the reform focused on, has never been studied. Filling this gap is the purpose of this research. This is important beyond the geographical boundaries of the kingdom since neighboring countries are also attempting the same substitution, yet there are no published accounts on how the workforce adjusts to this substitution.

Three linked research arteries inform this analysis within the 2014 to 2022 timeframe. The first focuses on the shifts in the number, density, and distribution of family physicians in the three reform stages and in the period between; the second concerns how the equity of the distribution within the phases, captured through density, dispersion, and the Gini coefficient, may have been impacted; and the third analyzes how the fourteen regional health units ranked over the years.

## 2 Methods

### 2.1 Study design

This study employs a retrospective, longitudinal panel design to assess the population-level and geographical distribution as well as equity of the family medicine workforce over a nine-year span (2014–2022) spanning the pre-reform phase of centralized planning (2014–2016), the reform phase (2017–2020), and the consolidation phase (2021–2022) of the three stages of the reform of the health sector in Saudi Arabia. Since the analysis uses a balanced panel, the same fourteen regional health administrative units and the same administrative level are observed in each year included in the study.

### 2.2 Data source

Data were sourced from the 2014 to 2022 Statistical Yearbooks published by the Saudi Ministry of Health (MOH) (30). The Yearbooks publish systematic national data on the health workforce and include annual population estimates as well as the number of family physicians by administrative unit. In line with MOH classifications, several large cities (Jeddah, Taif, Hafar Al-Batin, Bisha) are considered as separate health administrative units rather than being part of their respective administrative provinces. The present study maintains this classification in line with the source data and treats these as fourteen separate units as opposed to the thirteen administrative regions used in some other Saudi datasets. Only publicly available, aggregated data were used; no individual-level identifiers were accessed.

Scope of the workforce covered. The study is limited to family physicians working in the primary healthcare centres run by the Ministry of Health, together with the centres administratively attached to MOH hospitals. Family physicians who practise in primary care run by the private sector, by the military health services, or by university hospitals fall outside the data and are not counted. This boundary was chosen for two reasons. First, the question of interest is how the distribution of family physicians changed inside the MOH primary care network, which is the main public channel through which primary care is delivered in the Kingdom. Second, the MOH Statistical Yearbooks report the workforce in the same way for every region and every year of the series, which is what makes a longitudinal comparison possible. The excluded sectors are organised, staffed and managed under different arrangements, and no comparable region-by-year series exists for them. Pooling them with MOH figures would therefore mix data of uneven coverage and different organisational logic, and the regional and temporal estimates would no longer be comparable. The practical consequence, discussed in Section 4.2, is that every regional estimate in this paper refers to MOH provision only, and this matters most for the large urban units where non-MOH providers are concentrated. There are three reasons 2014 was chosen as the starting year for this study. First, it is prior to the initiation of the reform. Vision 2030 and the National Transformation Program were both announced in 2016. A series beginning in 2014 includes three complete years prior to the announcement of the reform. It also allows for the creation of a baseline that could not have been influenced by the reform. A shorter pre-reform period could mean the baseline would rest on one or two observations, and therefore, be vulnerable to an outlier year. Second, it is the first year where family medicine physicians are reported in all of the administrative units in the statistical yearbooks. Earlier years either report family medicine and general practice together or use administrative units that do not reconcile with the later years. Assuming backwards would not be necessary to extend the panel. Third, from 2014 on, the denominators are constant and population estimates at the unit level are published on a regular basis in all of the statistical yearbooks. Therefore, a comparable series becomes available, and the starting point is set far enough before the reform to be used as the baseline. It also does not rely on any particular policy event.

Because the reporting frame is not the same as the frame used by Saudi Arabia's civil administration, comments are warranted. The Kingdom is divided into 13 provinces, however, the Ministry reports 14 units for the health workforce. The two differ in two aspects. Four units, Jeddah, Taif, Hafar Al-Batin and Bisha, are separated from a larger province, and each is reported on separately. Jazan, Najran and Al-Baha are not included as separate units at this level in the workforce tables in this series. The implication for this analysis requires explanation: the four units are not comparable in size and the inequality indices account for the units in an equal manner, regardless of the size of their populations. For example, a unit of Bisha's size can show a significant change (even larger than a province of many millions of residents) that a unit of Bisha could not, and that change will be of equal importance to the index as the other province. This is the generally accepted manner in regional health-workforce writing, and it is appropriate to ask if the available administrative units are equal in the provision of resources, but this is inappropriate when the focus is on the number of affected people. Section 3.8 deals with what occurs to the outcomes when the four small city-level units are completely removed, and when each of the units is removed in succession.

### 2.3 Variables

The primary outcome measure was family physician density, being the number of qualified family medicine physicians per 10,000 population in the administrative unit and year. In this analysis, the population denominator for each of these was the MOH-published value for that unit and year. The reform phase was considered a categorical variable with the three levels: pre-reform, transformation, and post-reform.

### 2.4 Statistical analysis

Summary statistics of national and regional workforce indicators presented mean, median, range, and standard deviation of regional density for each year and phase. Regional inequality was assessed using three complementary measures: the Gini coefficient, Theil index, and coefficient of variation (CV). Relative dispersion is the focus of the Gini coefficient, while Theil index explicitly considers and highlights the contributions of extreme values. The CV is useful for considering the proportion of inequality in different time periods (43). Given the small number of regions, bootstrap 95% confidence intervals (1,000 resamples) were obtained for the Gini and Theil indices.

The temporal inequalities were analyzed by the Mann-Kendall test. The differences in the distribution of the reform phases were analyzed by the Kruskal–Wallis test, followed by the *post hoc* analysis pairwise comparisons of the Mann–Whitney *U* tests with the Bonferroni adjustment. Spearman's rank correlation was used for assessing the temporal stability of regional ordering.

The choice to work non-parametrically was tested rather than assumed. Regional density was tested for normality using the Shapiro–Wilk test and was assessed against a fitted normal using the Kolmogorov–Smirnov test, applied annually to the pooled panel and within each reform phase. Skewness and Excess Kurtosis were computed as well. The results are located in Table 1. Regarding the data, three arguments against parametric inference exist. The first is sample size. Considering the size of each of the fourteen observations in the annual cross-sections, a test of normality has little to no power, and, thus, a non-significant result is of little to no value. The second is shape. The pooled distribution exhibits right-skewness and departs from normality, per both tests (Shapiro–Wilk *W* = 0.901, *p* < 0.001; Kolmogorov–Smirnov *D* = 0.126, *p* = 0.033). Skewness, over the years, shifts from being very close to zero in the pre-reform years to being close to one in 2022, as the upper tail begins to elongate. The third is regarding the comparisons that matter most. Both distributions (pre-reform and post-reform) depart from normality, in the transformation phase (*W* = 0.936, *p* = 0.005) and in the post-reform phase (*W* = 0.913, *p* = 0.023), respectively. Concerning inequality indices rather than means, Gini, Theil and the Coefficient of Variation, have no tractable parametric sampling distribution at this sample size. Thus, their uncertainty is measured by bootstrap resampling, whereas classic sampling distribution is derived by closed-form. Rank-based tests and the bootstrap were preferred. Where a parametric equivalent exists, we ran it as a check and refer to it in Section 3.8 if our results differ.

**Table 1:** Tests of normality for regional family physician density, by year, pooled and by reform phase.

Sample	*n*	Shapiro–Wilk *W*	*p*	K–S *D*	*p*	Skewness	Excess kurtosis
2014	14	0.941	0.436	0.166	0.780	−0.22	+0.92
2015	14	0.986	0.996	0.119	0.976	−0.08	−0.61
2016	14	0.945	0.482	0.141	0.908	−0.33	−0.34
2017	14	0.949	0.538	0.131	0.944	+0.73	+0.42
2018	14	0.938	0.390	0.158	0.824	+0.70	+0.91
2019	14	0.946	0.497	0.139	0.916	+0.76	+0.56
2020	14	0.891	0.084	0.197	0.579	+0.73	−0.81
2021	14	0.881	0.060	0.170	0.751	+1.03	+0.16
2022	14	0.879	0.056	0.188	0.640	+1.00	+0.06
Pre-reform	42	0.969	0.314	0.105	0.709	−0.23	+0.30
Transformation	56	0.936	0.005	0.133	0.254	+0.86	+0.61
Post-reform	28	0.913	0.023	0.184	0.265	+0.98	+0.42
Pooled panel	126	0.901	<0.001	0.126	0.033	+1.32	+2.42

Since the administrative reclassification of physicians in 2021 introduced an artificial break in the series, the trend analyses were carried out in two forms. The artifact-adjusted form, in which 2021 is either omitted or modelled as a structural break, is treated as the primary estimate of the inequality trend; the full nine-year series is reported alongside it for completeness. The following analyses were used to examine how much of the main finding depends on this reclassification. Three analyses were performed. The first analysis is a recalculation of all indices and all trend tests for the eight-year time series with 2021 omitted. The second analysis constructs a structural break and analyzes 2020 and 2022 directly and also performs a test of the 2014–2020 time series. The third analysis performs a phase comparison with 2022 post reform. Similar to the first analysis, a unit-grain sensitivity analysis was performed by recomputing all indices limited to the ten provincial units after the removal of the four city units. Also, a leave-one-unit-out analysis of the 2022 cross section was performed to find a unit that could potentially drive the result. All the results have been reported in Sections 3.7 and 3.8, with additional results providing indices and trend tests in Tables 2, 3.

**Table 2:** Sensitivity analysis: inequality trends with and without the 2021 administrative artifact.

Series	Index	First year	Final year	Change (%)	Mann–Kendall *S*	*Z*	*p*
Full series 2014–2022 (*n* = 9)	Gini	0.160	0.210	+31.2	20	1.98	0.048
	Theil	0.051	0.071	+39.2	16	1.56	0.118
	CV	0.316	0.406	+28.5	20	1.98	0.048
Excluding 2021 (*n* = 8)	Gini	0.160	0.210	+31.2	14	1.61	0.108
	Theil	0.051	0.071	+39.2	10	1.11	0.266
	CV	0.316	0.406	+28.5	14	1.61	0.108
Pre-break 2014–2020 (*n* = 7)	Gini	0.160	0.157	−1.9	7	0.90	0.368
	Theil	0.051	0.039	−23.5	3	0.30	0.764
	CV	0.316	0.298	−5.7	7	0.90	0.368
Structural-break contrasts and phase comparison excluding 2021.							

Index	2020 (clean year)	2022 (clean year)	Change (%)
Gini	0.157	0.210	+33.8
Theil	0.039	0.071	+82.1
CV	0.298	0.406	+36.2
Phase-level inequality with the post-reform phase represented by 2022 alone (Kruskal–Wallis *H* = 22.58, df = 2, *p* < 0.001).			

Phase	Mean density	Mean Gini	Mean Theil	Mean CV
Pre-reform (2014–2016)	1.13	0.140	0.036	0.266
Transformation (2017–2020)	1.48	0.168	0.046	0.320
Post-reform, 2022 only (2021 excluded)	1.91	0.210	0.071	0.406
Post-reform as published (2021–2022)	1.61	0.231	0.087	0.448

**Table 3:** Robustness to the definition of the administrative unit.

Unit definition	Gini 2014	Gini 2022	Gini change (%)	Theil 2014	Theil 2022	CV 2014	CV 2022
All 14 MOH health administrative units	0.160	0.210	+31.2	0.051	0.071	0.316	0.406
10 province-level units (4 city-level units removed)	0.129	0.184	+43.4	0.029	0.054	0.257	0.348
Leave-one-unit-out influence on the 2022 Gini coefficient (full-sample Gini = 0.210).							

Unit excluded	Gini 2022 without unit	Difference from full sample
Bisha	0.178	−0.032
Riyadh	0.202	−0.008
Northern Borders	0.204	−0.006
Jeddah	0.206	−0.005
Eastern	0.208	−0.002
Makkah	0.208	−0.002
Tabuk	0.210	+0.000
Qassim	0.211	+0.001
Hafar Al-Batin	0.214	+0.004
Hail	0.215	+0.005
Al-Ahsa	0.215	+0.005
Asir	0.217	+0.007
Madinah	0.218	+0.007
Taif	0.217	+0.007

To identify the regional trajectory typology, hierarchical cluster analysis (Ward's method) was performed on z-standardized regional density trajectories. *k* = 3 was selected based on dendrogram interpretation as well as the interpretability of the clusters. A trajectory for each region spanning the study period was summarized with a linear regression model.

Mean density and percentage change data sets were analyzed by phase using choropleth mapping.

All analyses were performed in Python 3.12 using the NumPy, SciPy, pandas, and scipy.cluster libraries.

### 2.5 Ethical consideration

Ethical approval was not required because the study used only publicly available, aggregated administrative data that contain no individual-level or identifiable patient information. The Statistical Yearbooks are published openly by the Ministry of Health for unrestricted public use, and the analysis involved no human participants, no clinical intervention, and no access to medical records. Secondary analysis of fully anonymised, aggregated public data of this kind therefore falls outside the scope of institutional review board approval.

## 3 Results

### 3.1 Expansion of the national family medicine workforce

From 2014 to 2022, the total number of family physicians in Saudi Arabia increased from 2,625 to 4,273, by the addition of 1648 or 62.8% of physicians. Over the same time, the resident population increased from about 27.4 million to 29.0 million, so the increase in physicians out paced the increase of the population, and the national density went from 0.96 to 1.47 physicians per 10,000 people, a relative increase of 53.0%. The increase was not linear. Figure 1 shows the rise of the national family physician density during the transformation phase to a peak of 1.58 per 10,000 in 2020 and a sharp decrease to 2021 to 1.02, and then to 2022 to 1.47. The 2021 decrease is likely not an absolute loss of the workforce, rather it is likely a structural reclassification of the physicians as a result of the migration of services to the regional health clusters during the implementation of Vision 2030. The 2021 observation is therefore treated as an administrative artifact rather than a real change in supply, and the trend results in Sections 3.3 and 3.7 give precedence to estimates that exclude it or model it as a structural break. Table 4 shows the national changes along with the changes that occurred at the regional level.

**Figure 1:**
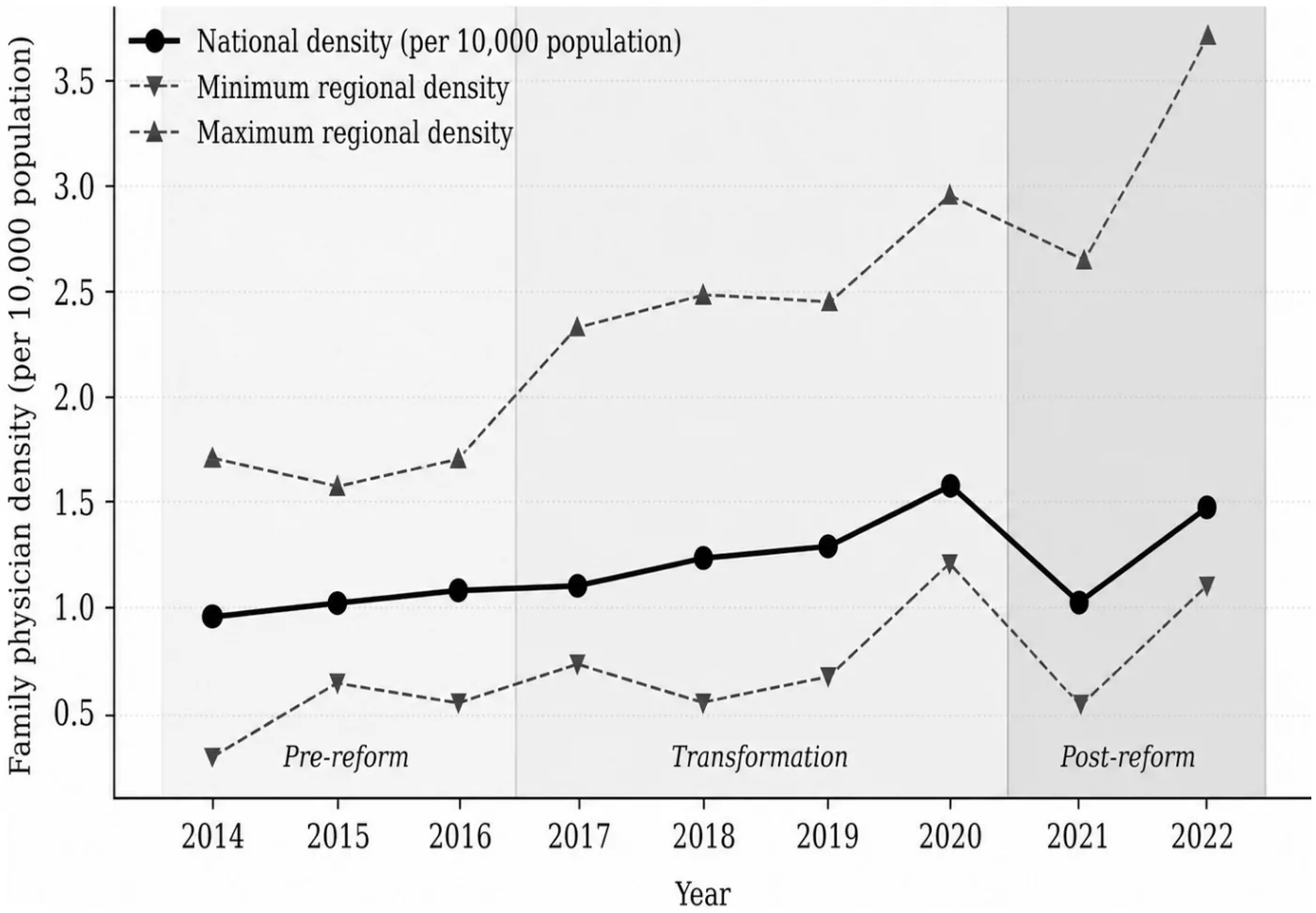
National family physician density and regional extremes, 2014–2022.

**Table 4:** National and regional distribution of family physicians and population, 2014–2022.

Year	Total physicians	National density (per 10,000)	Min regional density	Max regional density	Range	SD
2014	2,625	0.96	0.29	1.71	1.42	0.33
2015	2,867	1.02	0.64	1.57	0.93	0.25
2016	3,067	1.08	0.55	1.70	1.15	0.32
2017	3,217	1.11	0.73	2.34	1.60	0.42
2018	3,696	1.24	0.55	2.49	1.93	0.46
2019	3,958	1.29	0.67	2.46	1.79	0.45
2020	4,948	1.58	1.21	2.96	1.75	0.56
2021	3,088	1.02	0.54	2.65	2.11	0.64
2022	4,273	1.47	1.10	3.72	2.61	0.78

### 3.2 Longitudinal trends in regional density

From 2014 to 2022, mean regional density increased from 1.05 to 1.91 per 10,000, representing a national 82.4% increase. This outpaced the 53.0% growth in the national, population-weighted density. The increase in the mean regional density, as compared to the growth of the most populous regions, shows that the smaller and less populous regions experienced relatively larger gains, on average. This was confirmed by the Mann-Kendall test where statistically significant monotonic increases in mean regional density were noted for this time period (*S* = 28, *z* = 2.82, *p* = 0.005). Individual regional densities increased at very different rates. An example is Bisha where mean regional density increased faster at 0.36 units per year with a high degree of confidence (*p* < 0.001), compared to a negative growth of 0.01 units per year for both Hafar Al-Batin and Jeddah, and Hail (Table 5). Thus regional growth was concentrated with very rapid growth nested among very stagnant regions. Table 5 shows that the regions that achieved the highest relative gains from the health reform began with the lowest levels prior to the reform. Before the reform, Bisha recorded a density of 0.88 per 10,000, and after the reform, recorded a density of 3.18 per 10,000, a relative gain of 262.1%. The Northern Borders region, before the reform, had a density of 1.22 per 10,000, and after the reform, had a density of 2.73 per 10,000, a gain of 122.9%. The other regions that recorded gains were Madinah (82.6%), Tabuk (56.3%), Qassim (44.7%), while Jeddah, Makkah, Al-Ahsa, Hail, and Hafar Al-Batin all recorded gains of less than 30% after the health reform. These latter regions recorded densities of family physicians at or below the levels before the health reform, despite participating in the national health reform. As shown in Figure 2, three different regional density patterns of family physicians were observed (shown in different colors). The most peripheral regions recorded the greatest gains. The redistribution was therefore from urban or near-urban to the more peripheral regions. Figure 3 shows family physicians' density in the various regions at different stages in the health reform.

**Table 5:** Regional family physician density by reform phase and percentage change, 2014–2022.

Region	Pre-reform 2014–2016 (mean)	Transformation 2017–2020 (mean)	Post-reform 2021–2022 (mean)	Absolute change	Percentage change (%)
Bisha	0.88	2.49	3.18	+2.30	+262.1%
Northern Borders	1.22	1.73	2.73	+1.50	+122.9%
Madinah	0.89	1.34	1.62	+0.73	+82.6%
Tabuk	1.39	1.67	2.17	+0.78	+56.3%
Qassim	1.37	2.04	1.98	+0.61	+44.7%
Taif	1.05	1.21	1.46	+0.41	+38.8%
Asir	1.28	1.51	1.74	+0.46	+35.7%
Eastern	0.90	1.14	1.15	+0.24	+26.7%
Riyadh	0.86	1.41	0.99	+0.13	+15.1%
Makkah	1.01	1.12	1.01	−0.01	−0.5%
Al-Ahsa	1.06	1.13	1.03	−0.03	−2.5%
Jeddah	1.02	0.79	0.90	−0.12	−11.5%
Hail	1.55	1.62	1.36	−0.19	−12.4%
Hafar Al-Batin	1.38	1.56	1.21	−0.18	−12.9%

**Figure 2:**
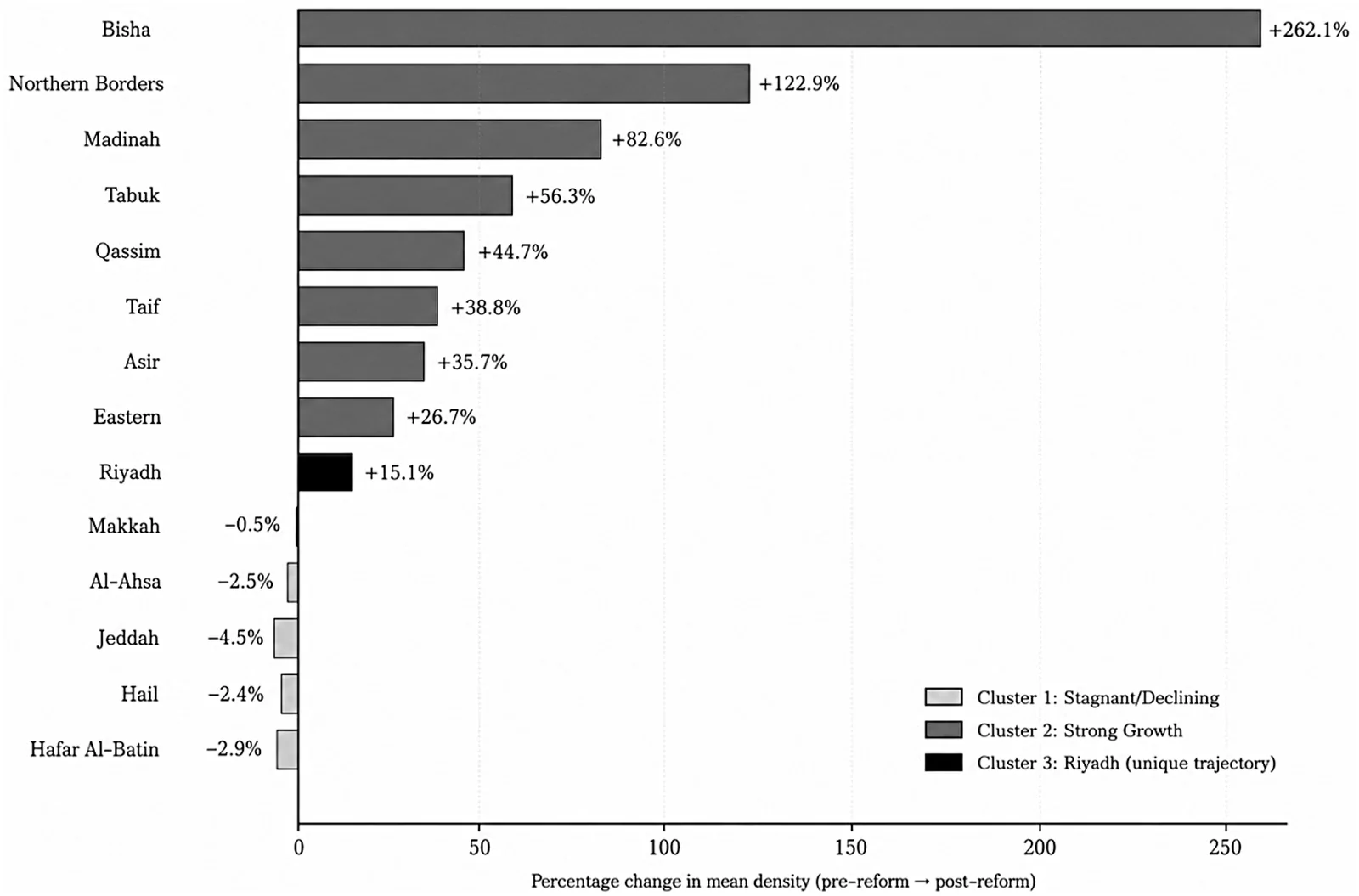
Regional family physician density change by reform phases, colour-coded by trajectory cluster (see Section 3.5).

**Figure 3:**
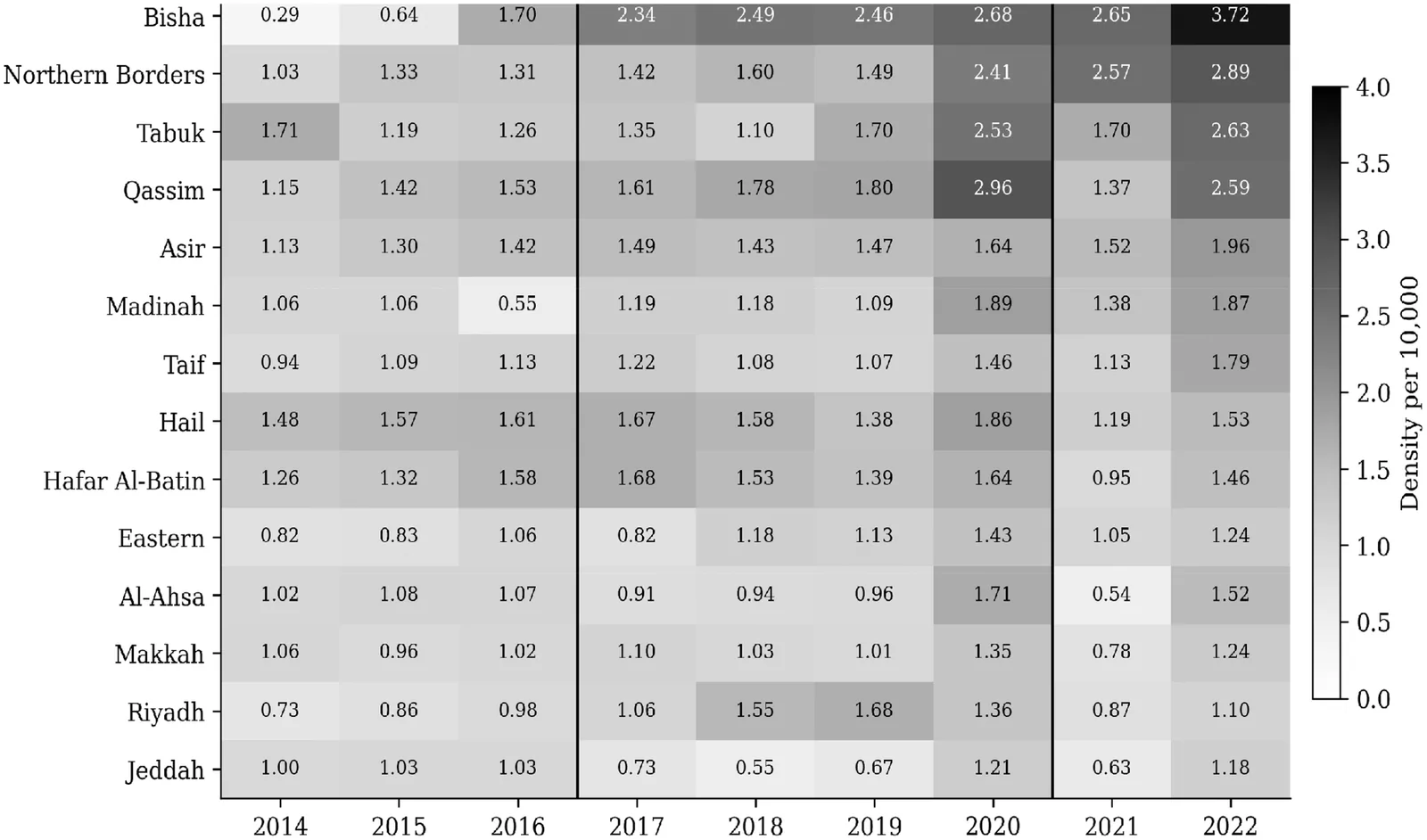
Heatmap of family physician density by region and year, with reform phase boundaries.

### 3.3 Inequality dynamics: gini, theil, and coefficient of variation

All three inequality measures were higher at the end of the study period than at the start, although, as shown below and in Section 3.7, the year-on-year trend is marginal and depends on the 2021 observation. As shown in Table 6, the Gini coefficient rose from 0.160 (bootstrap 95% CI 0.071–0.243) in 2014 to 0.210 (95% CI 0.131–0.248) in 2022, a relative increase of 31.5%. The Theil index rose from 0.051 to 0.071 (+39.1%), and the coefficient of variation rose from 0.316 to 0.406 (+28.6%). The Mann–Kendall test indicated a monotonic increase of borderline significance in the Gini coefficient (*S* = 20, *z* = 1.98, *p* = 0.048) and the coefficient of variation (*S* = 20, *z* = 1.98, *p* = 0.048); the trend in the Theil index was in the same direction but not significant (*S* = 16, *z* = 1.56, *p* = 0.118). In the artifact-adjusted series with 2021 excluded, which is the primary trend estimate, neither the Gini nor the coefficient of variation trend reached significance (*S* = 14, *z* = 1.61, *p* = 0.108; Table 2). The monotonic trend should therefore be read as suggestive rather than established, whereas the endpoint increase between 2014 and 2022 does not depend on 2021.The Lorenz curves in Figure 4 visualise this divergence: the curve for the post-reform phase lies furthest from the line of perfect equality, while the pre-reform curve is the closest to it.

**Table 6:** Inequality indices and bootstrap 95% confidence intervals, 2014–2022.

Year	Gini	Gini 95% CI	Theil	Theil 95% CI	CV
2014	0.160	0.071–0.243	0.051	0.009–0.112	0.316
2015	0.123	0.077–0.158	0.024	0.010–0.040	0.226
2016	0.137	0.084–0.188	0.032	0.012–0.062	0.256
2017	0.168	0.095–0.215	0.045	0.016–0.076	0.317
2018	0.175	0.097–0.241	0.053	0.016–0.098	0.340
2019	0.170	0.097–0.221	0.047	0.017–0.081	0.325
2020	0.157	0.096–0.179	0.039	0.017–0.052	0.298
2021	0.252	0.142–0.308	0.103	0.035–0.157	0.490
2022	0.210	0.131–0.248	0.071	0.029–0.104	0.406

**Figure 4:**
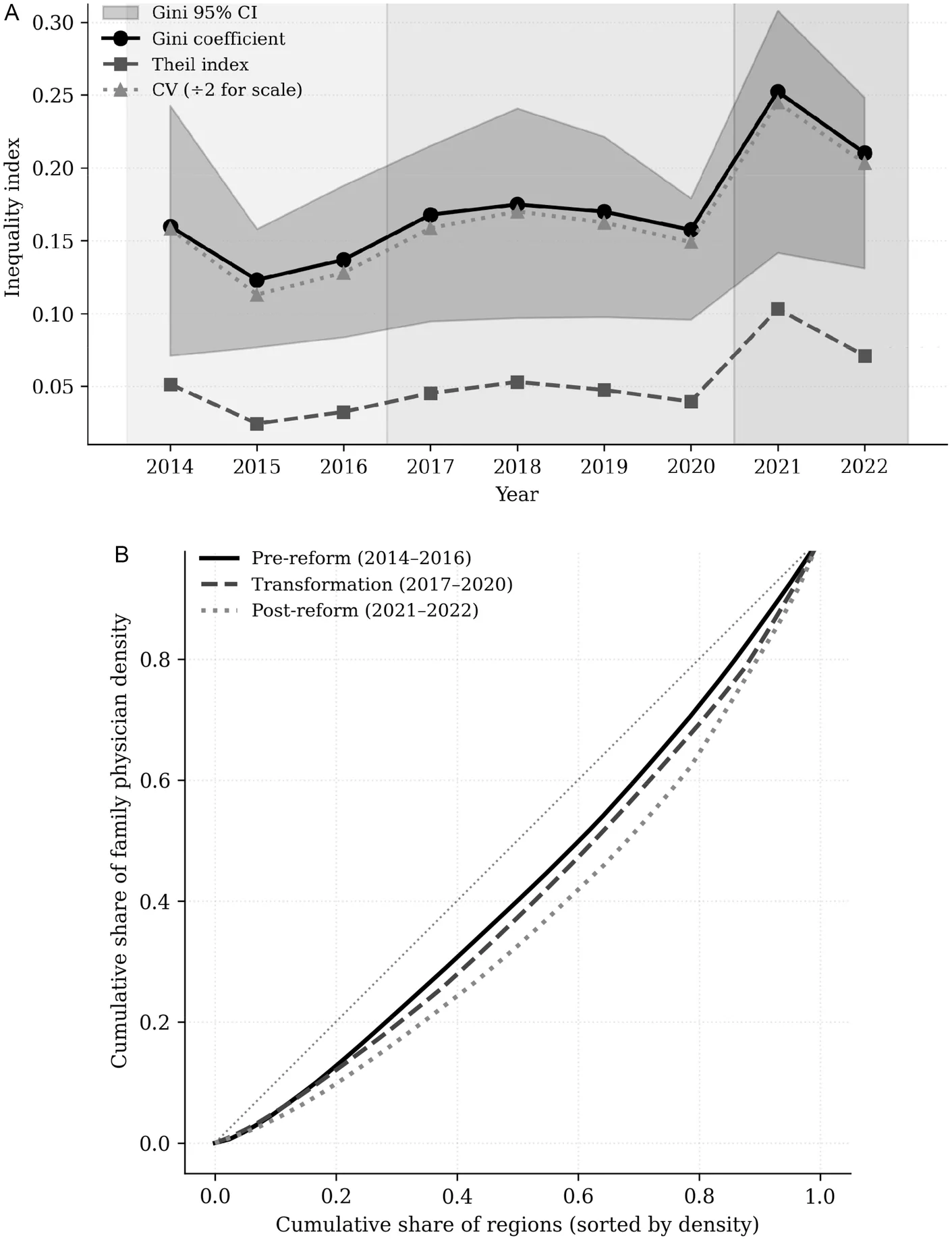
**(A)** Trends in three inequality measures of family physician distribution with bootstrap 95% confidence intervals. **(B)** Lorenz curves for regional family physicians distribution by reform phase, with diagonal reference lines.

The CV-track confirms the Gini and Theil findings while being insensitive to distributional assumptions, and the agreement of all three measures on the direction of change offers some reassurance that the endpoint widening is not an artefact of one particular index, even though the monotonic trend itself is not robust. The widening of confidence intervals after 2020 also reflects the entry of extreme regional values (Bisha, Northern Borders) into the distribution, which is itself a feature of the reform pattern rather than statistical noise.

### 3.4 Phase-Level comparisons

When looking at regional density by reform phase, we see an increase from 1.13 per 10,000 in the pre-reform phase to 1.48 in the transformation phase and to 1.61 in the post-reform phase. The Kruskal–Wallis Test showed that the three phases were significantly different (*H* = 15.21, df = 2, *p* < 0.001). Pairwise Mann–Whitney tests, with the Bonferroni adjustment, confirmed that the transformation and post-reform phases both significantly differed from the pre-reform phase (p_adj = 0.001 and p_adj = 0.016, respectively), indicating that the major phase of regional density increase occurred in the transformation phase and was sustained, but not further increased, in the post-reform phase. The inequality phase patterns ran in the opposite direction. Phase-mean Gini increased from 0.140 (pre-reform) to 0.167 (transformation) to 0.231 (post-reform). While phase-mean Theil increased from 0.036 to 0.046 to 0.087 and phase-mean CV increased from 0.266 to 0.320 to 0.448. Therefore, each subsequent phase increased mean density, but at the cost of a more unequal distribution of that density (Figure 5; Table 7).

**Figure 5:**
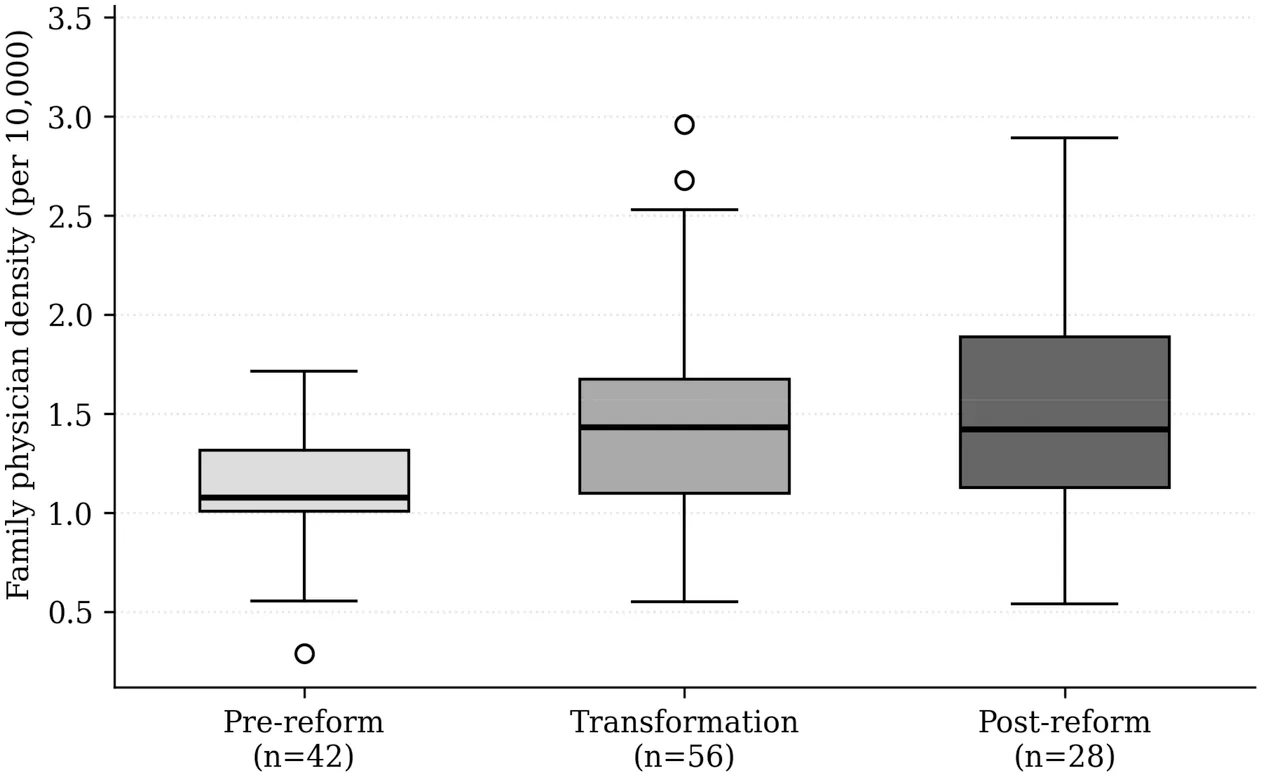
Boxplots of regional family physician density by reform phase, showing the interquartile range; and medians.

**Table 7:** Phase-level density and inequality indices.

Phase	Years	Mean density (per 10,000)	Mean Gini	Mean Theil	Mean CV
Pre-reform	2014–2016	1.13	0.140	0.036	0.266
Transformation	2017–2020	1.48	0.167	0.046	0.320
Post-reform	2021–2022	1.61	0.231	0.087	0.448

Cross-phase tests confirming differences in inequality index values (with aggregate values across phases) supported these patterns. Pooled phase-level Gini values were 0.146 (pre-reform), 0.186 (transformation), and 0.251 (post-reform). These results showed that the post-reform phase Gini value was approximately 71% greater than that of the pre-reform phase.

### 3.5 Spatial patterns and regional trajectory clusters

Figure 6 highlights the mean density with the study period. This shows high density with a central south concentration having Bisha (mean 2.11 per 10,000), Qassim (1.80), and Northern Borders (1.78). Large municipalities, such as; Jeddah (0.89), Makkah (1.05), and Riyadh (1.13), are located along the western seaboard, and have much lower density despite a large population and well-developed municipal services. The percentage-change map (Figure 7) reveals clearer logical reasoning for the reform. It shows that the southern and central regions along with the northern periphery achieved growth, simultaneously, the western corridor was the site for diminishing growth or no growth. Within the MOH workforce, and counter to the conventional urban-bias pattern, growth concentrated in the less populated peripheral regions while the urban western corridor stagnated. Because non-MOH providers are concentrated in exactly these urban units (Section 4.2), this contrast describes MOH provision and not total family physician supply. The standardized regional trajectories allowed for certain interpretations through Ward (hierarchical) clustering.

**Figure 6:**
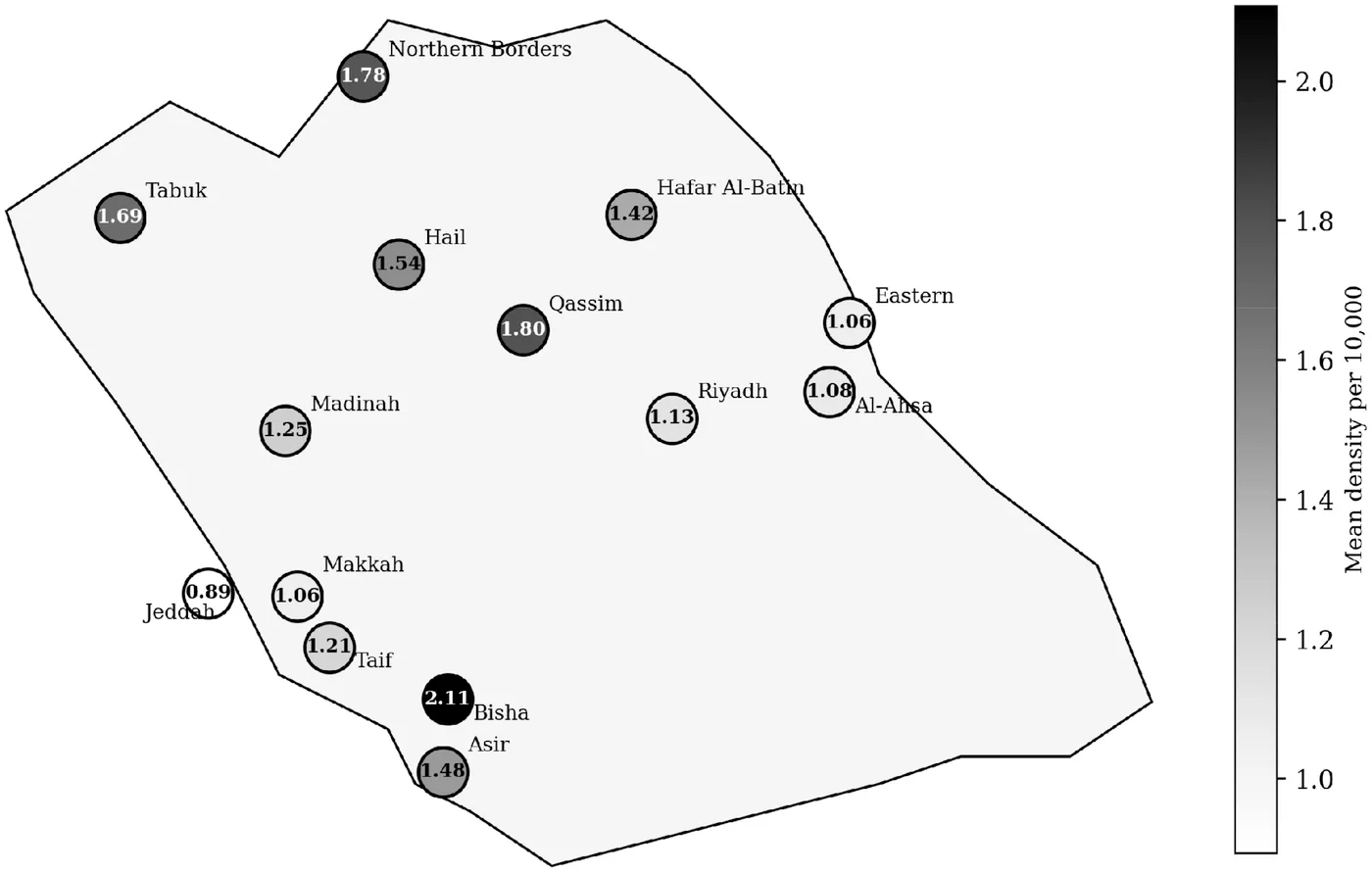
Bubble map of mean family physician (per 10,000 population) by health administrative unit,2014–2022.

**Figure 7:**
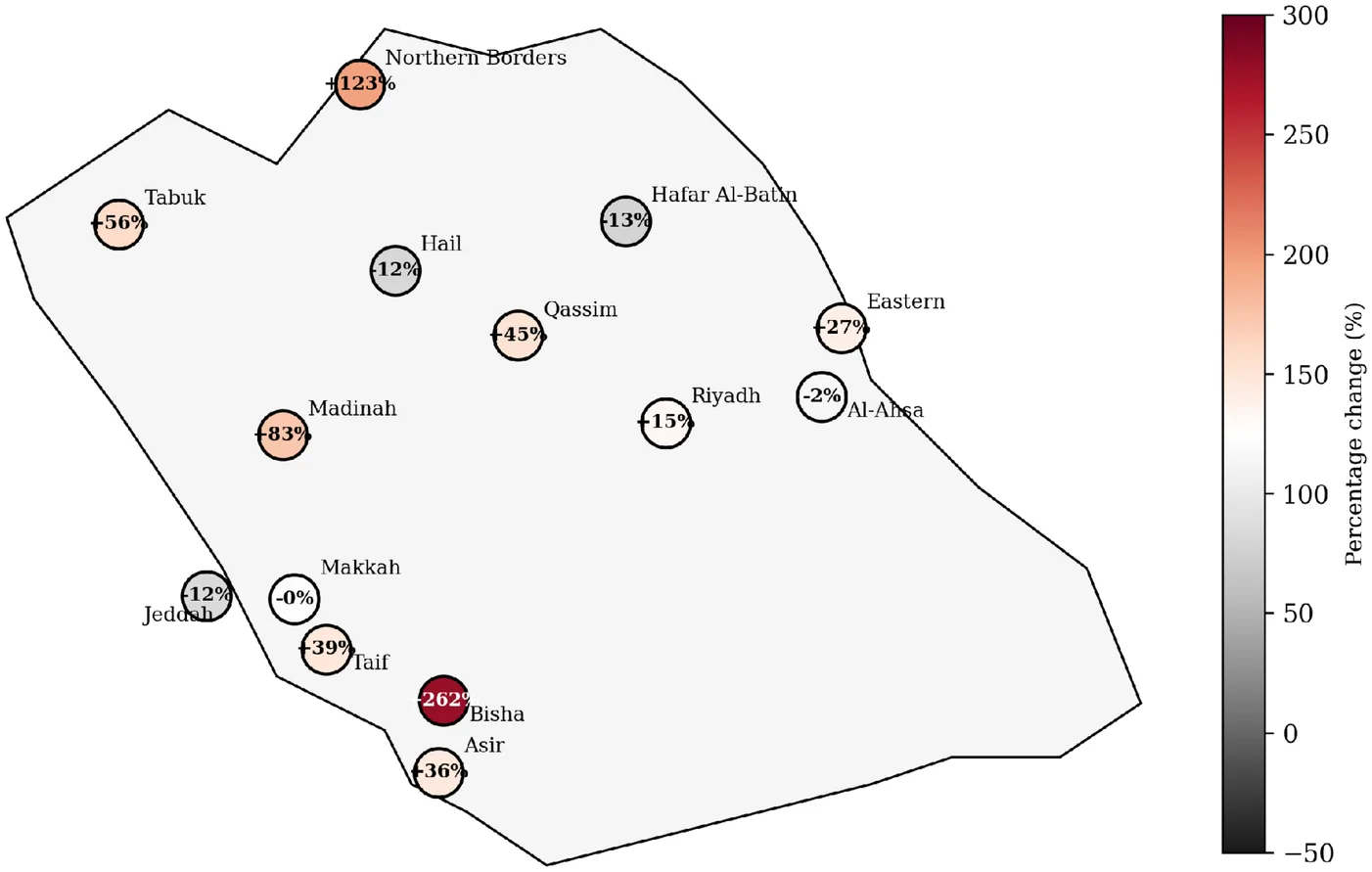
Choropleth map of family physician density change between the pre-reform and post-reform phases, Red gains, grey declines.

Cluster 1 includes Jeddah (−11.5%), Makkah (−0.5%), Al-Ahsa (−2.5%), Hail (−12.4%), and Hafar Al-Batin (−12.9%). Cluster 2 includes Bisha (+262.1%), Northern Borders (+122.9%), Madinah (+82.6%), Tabuk (+56.3%), Qassim (+44.7%), Taif (+38.8%), Asir (+35.7%), and Eastern (+26.7%). Cluster 3 contains Riyadh, the only case in which the peak of the transformation phase was at 1.41 per 10,000, falling to 0.99 during the post-reform phase, representing a + 15.1% positive change. The Riyadh case is more strongly justified by the supply-side shocks during the workforce reclassification of 2021 than by a sustained reform-induced trend.

### 3.6 Regional rank stability over time

Using Spearman rank correlation on regional density helps analyze changes in regional ordering during the period of reforms. From 2014 to 2022, the correlations were weak (*ρ* = 0.21, *p* = 0.46), which suggests many changes in the regions that were at the top and the bottom during this time period. However, for shorter time spans, the rank correlations showed more stability (2020–2022: *ρ* = 0.90, *p* < 0.001; 2017–2022: *ρ* = 0.65; *p* = 0.011). This suggests that the main changes in regional ordering occurred during the reform process, and not afterward. By 2022 most of the regional ordering had settled toward the “peripheral winners” and the “urban laggards” and this ordering showed stable after 2020.

### 3.7 Sensitivity analysis: the 2021 reclassification

Removing 2021 leaves the endpoint widening unchanged but removes the statistical significance of the monotonic trend, so the artifact-adjusted estimates reported here are the ones on which the trend claim should rest. With 2021 being an interior point, the endpoints of the series remain the same upon its removal. The Gini coefficient ranges from 0.160 in 2014 to 0.210 in 2022, the Theil index from 0.051 to 0.071, and the coefficient of variation from 0.316 to 0.406. Thus, relative increases published in Section 3.3 are maintained in the same form. As for the Mann–Kendall test. For a period without 2021 a monotonic (increasing) trend is not significant for either the Gini or the coefficient of variation (*S* = 14, *Z* = 1.61, *p* = 0.108). The Theil index is not significant in either case (*p* = 0.118 and *p* = 0.266). Therefore, the statistical significance of the monotonic trend rests on the 2021 observation, and the trend is best described as suggestive rather than robust. The claim of increased inequality at the end of the period compared to the beginning of the period does hold even without 2021.

modeling 2021 as a structural break makes the data more clear rather than less clear. Looking at the unaffected years on either side of 2021, we see that the Gini increased from 2020 to 2022, rising by 33.8% (from 2020: 0.157 to 2022: 0.210), and Theil and Coefficient of Variation increasing by 82.1% and 36.2% respectively. In the 2014–2020 sub-series, we actually see a decrease in the overall levels of inequality: the Gini begins and ends this sub-series at 0.160 and 0.157 respectively (-1.9%), while mean regional density has increased by about 78% (Mann–Kendall *Z* = 3.00, *p* = 0.003). The rising levels of inequality in 2022 and 2021 are not a gradual drift of inequality, they are concentrated in the final two years of this study, particularly in the years of the building of the health clusters and the pre-2020 period which was actually neutral with respect to inequality. Looking at 2022 again in the post-reform phase, we observe phase average Ginis of 0.140, 0.167 and 0.210, while including 2021 yields a Kruskal–Wallis statistic that is larger, rather than smaller (*H* = 22.58, *p* < 0.001). The full results are available in Table 2. Two main things can be concluded. Without considering 2021, the main conclusion of the paper remains the same, and the divergence occurs during the building of the clusters, rather than during the earlier phases of the health reform.

### 3.8 Robustness to administrative-unit definition

It is unlikely that small, city-level units are responsible for the reported inequalities. Restricting the analysis to the ten province-level units, and thus removing Jeddah, Taif, Hafar Al-Batin and Bisha, shows that the Gini increased from 0.129 in 2014 to 0.184 in 2022, representing an increase of 43.4%. This increase is larger than the increase observed in the full set of fourteen units, with both the Theil index and the Coefficient of Variation showing an upward trend. Removing the small units from the analysis shows a decrease in measured inequality. However, in this case, removing the small units has increased the slope of the trend. Applying the leave-one-unit-out method for the years 2014 and 2022, indicates that Bisha exerts a strong positive influence over the slope for 2022. In 2022, removing Bisha reduces the Gini index from 0.210 to 0.178. Excluding all other units only changes the Gini index by up to 0.008. For fourteen of the units, the change in the Gini index is less than or equal to 0.005. Thus, the omission of Bisha for 2022 shows a more unequal distribution compared to 2014. Table 3 illustrates the two modifications. Readers should also be cautious about the absolute values since the equal weighting convention adopted in the methods is relative to a unit weighting scheme based on population, which would place more influence on Riyadh and the Eastern Province and less on Bisha.

## 4 Discussion

The distribution of the MOH-employed family medicine workforce before and after the primary care reform in Saudi Arabia was described. The design is observational and uncontrolled, so the changes reported here are concurrent with the health reform rather than attributable to it. The analysis identified two major trends. Analysis has shown the rapid growth and uneven nature of the family medicine workforce. From 2014 to 2022 nationwide growth in family medicine physicians increased by 53% and was accompanied by the addition of 1,600 physicians. Over the same period a growth in family medicine physician density was observed, as was an increase in the inequitable distribution of family medicine physicians, as measured by the Gini, Theil, and the coefficient of variation. The agreement of the three indicators on the direction of change supports a real increase in inequality between the two endpoints of the series, although the year-on-year trend is marginal and not robust to the exclusion of 2021. During this time family medicine accounted for approximately 10% of the total physician workforce at a density of 0.03 family physicians per 1,000. Using comparable measures the family medicine physician workforce has grown, and is approaching the family medicine benchmark at the national and regional levels when compared to the family medicine national benchmark.

The finding that stands out is the direction of the uneven change within the MOH workforce. It has to be read with one caveat in mind from the start: the data cover Ministry of Health family physicians only, and the private, military and university providers that are left out are concentrated in Riyadh, Jeddah, Makkah and the Eastern Province, which are the very units that appear stagnant here (see Sections 2.2 and 4.2). Urban bias is highly documented in published health workforce literature. While Wang et al. (44) found bias in China's eastern zones, and other researchers found a steady rural bias in the US (45) and greater socio-economic bias in primary care in England (46), the MOH family medicine workforce in Saudi Arabia appears to have moved against the prevalent trend during the health reform period. More absolute growth was recorded in the periphery, where Bisha (+262%) and Northern Borders (+123%) recorded growth along with Madinah, Tabuk, Qassim, Taif, Asir and the Eastern Province, while Jeddah, Makkah, Al-Ahsa, Hail and Hafar Al-Batin, stagnated or recorded decreases in the ministry-employed workforce. Within the MOH workforce, this looks like a periphery-favouring pattern that differs from the urban bias usually described in the international literature. Whether it amounts to a genuine reversal of urban bias cannot be settled with these data, since a family physician who moves from an MOH centre to a private or university clinic in the same city disappears from the series. What can be said is that, for MOH provision, growth was larger the further a unit was from the metropolitan centres, while the metropolitan units stagnated.

This pattern is consistent with the Saudisation Programme and the capacity-building initiatives of Vision 2030 having had their most visible effect on the fringes (7, 12), although the design cannot attribute the change to them. Almost all family medicine residencies have been in the major metropolitan cities. The rapid development of residency programs in the Northern Borders and Southern Region shows that the development of new paths and incentives focused on the periphery was the first to be addressed. Whether this peripheral development of family medicine residency programs constituted a strategic placement of Saudi family physicians is beyond the scope of this research. Al-Ghamdi et al., and the strategic framework of Al-Khaldi et al., anticipated diverse residency expansion. The value of this longitudinal data reveals that the periphery was developed before the urban centers (14, 31).

The drop in 2021 is worth noting. A sharp drop in the national concentration, from 1.58 to 1.02 per 10,000 citizens, with a subsequent increase to 1.47 in 2022, indicates an ongoing labor force issue. Possibly, the movement of workforce to the Vision 2030 health regional clusters, as well as the restructuring of the health workforce's administrative categories, explains this loss (9, 16). This is likely a reclassification of the workforce, meaning it could be a net loss; however, it may actually increase the range of work force and inequality post-primary care reform. Policy must consider the 2021 to 2022 workforce gap. From the sensitivity analyses in Section 3.7, the direction and magnitude of the endpoint widening remain when 2021 is removed, but the Mann–Kendall trend loses its significance (*p* = 0.108). This is why the artifact-adjusted estimates are treated as primary, and why the monotonic trend is presented as suggestive rather than established. The concentrated divergence of 2020 and 2021 would also remain.

In regard to Riyadh, the capital, the transformation phase recorded a peak of 1.41 per 10,000 citizens, whereas the post-primary care reform phase recorded a dip to 0.99, resulting in a positive, 15.1% difference. The other 13 units did not record this movement. One way to interpret this is that the reclassification of 2021 to 2021 had a significant impact on the largest workforce center, thus a large portion of this decline is spurious.

Another idea is whether people moving from the capital have helped populate areas further away from the capital. Of course, this could be happening simultaneously. Neither is comforting to a capital which is due to grow in order to fulfill the “family physician for every family” program (15, 32).

Widening inequality in Saudi Arabia isn't unique. Boniol et al. (47), observed that, when conflicts exist between universal health coverage and geographic constraints, increases in global health workforce stocks may be accompanied by growing inequities. Unlike many other countries, the conflicts associated with the Saudi Arabian health system may be more complex. As outlined in the preceding sections, in contrast to most health system reforms, which increase inequities by concentrating the benefits in already served urban areas, the MOH family physician workforce recorded its greatest relative growth in density in the geographically rural and remote areas, while the major urban centers (which have well-developed primary health care and are serviced by both the Ministry of Health and non-Ministry actors), had a lesser relative growth in the ministry-employed workforce. This may lead to inequities in service availability. For example, if private, more advanced, and better health care services exist in Jeddah and Makkah, compared to Bisha, then Bisha's residents will be able to access health care services that are not available to the residents of Jeddah and Makkah. Conversely, if this is not the case, then the economic burden will fall on the poor urban families and chronically ill patients who require a continuous primary care service.

The timing of this case invites a reading of decentralization outside of purely clinical policy. On the decision-space account, the delegation of authority to the health clusters considerably increased local discretion regarding the organization of services and the deployment of labor (11, 25, 33). Thus, clusters were able to reorganize services and move staff well prior to being able to compete for them. The ability to exercise the discretion that was granted varied. Clusters comprising old, established academic and metropolitan institutions began with managerial infrastructure that newly constituted peripheral clusters lacked. Kolehmainen-Aitken's observation that staffing equity is among the first casualties when administrative authority is extended beyond the limits of the human-resource capability fits the context described here and is therefore worth noting (28). The case study of Tunisia reported by Giusti and Persiani is the most relevant in this regard. Decentralization was intentionally used to address health inequities resulting from disparities across geographic areas; the authors show that a uniform national model did not work, different regions had different pre-existing conditions prior to the reform, and the actual implementation of the decentralization policy varied based on the regions' needs and was organized by national protocols (34). The authors' description is of a multi-speed implementation that, for a time, resulted in greater rather than lesser differentiation. This description appears to capture the case of Saudi Arabia also. There is, however, a caveat.

The counter-intuitive nature of these findings is due to the fact that it is the peripheral regions that have shown the highest levels of change. It is unclear whether the Tunisian analysis will hold true and the slow-moving clusters will eventually catch up and diminish the divergence, or whether the divergence will remain. This cannot be addressed using a series spanning only two years of the transition to clusters. This is an empirical concern for the 2023- 2025 yearbooks.

These findings also corroborate a number of recent studies on the Saudi PHC system on a regional level, although some studies have examined different variables. Al-Sheddi et al. (19) found that between 2017 and 2021 there was a regional disparity in the distribution of primary health care centers. Kattan (22) and Kattan and Alshahrani (23) found similar distributions in the health care facilities in 2022 and the total physician workforce respectively, and Qattan and Kattan (24) found a similar distribution among obstetrician–gynaecologists. The comparative advantage of the present study is that it takes a longitudinal and multi-dimensional perspective on family medicine for the entire 2014–2022 period, compared to a single year focus. The fact that separate studies have documented similar issues is also important.

Some practical implications are evident at this level. The “gatekeeper” model of family medicine relies on a community-based presence as opposed to being simply situated in a segment of a system. Without a community presence, patients will skirt primary care and will be referred immediately to tertiary care, a phenomenon documented in many family medicine systems globally. While telemedicine has benefits, it has been positioned as a substitute for primary care in underserved areas. It will be of limited value in lieu of a primary care system that is embedded in the community for the management of chronic disease and the maintenance of care, which accounts for the bulk of family medicine (48). Residency training outside of major urban centers, tailored regional retention strategies, and closer assessment of the “urban stagnation cluster” are warranted next. Alshaharni outlines pragmatic family medicine challenges in the Aseer region of Saudi Arabia. These likely typify the constraints facing family medicine in the region. Addressing constraints will be a better investment of family medicine resources than additional workforce efforts. Similar views from a more distal perspective reinforce this position—monitoring equity will be of greater concern than additional workforce quotas (35–37).

Whether Saudi primary care requires a compulsory referral system is a question without a single answer, and the ambiguity relates to the unequalness analyzed here. The family medicine model that underlies the reform has a formal gatekeeping function. Patients attach to a primary care team, contact is to be made there, and, by referral, access to specialty and hospital care is to be mediated. An electronic referral system provides the necessary administrative system (6, 11, 15). In actual fact, the gate is more of a gap. Presenting to hospital outpatient clinics and emergency departments is still common. Not all primary care clinics have a stated patient-attached physician, and how much the gate is enforced depends on the capacity of a given primary care clinic (6, 10, 32). The effect on equity is not neutral, and it is in the direction that local statistics on family physicians would not indicate. A requirement for a referral makes the local family physicians a system-wide constraint. Where the gate is enforced and physicians are scarce, the shortage of physicians is experienced in a delay of specialist care. Where the gate is enforced and physicians are abundant, the same rule increases the coordination of care at no additional cost. With a soft gate, the opposite is true.

This bypasses the shortage in underserved regions. Unfortunately, it does it by placing these patients on hospital services and only allowing them to those who live close to hospitals, which is often urban residents and the wealthier (38–40). The regions that we found as stagnant (Jeddah, Makkah, Al-Ahsa, Hail and Hafar Al-Batin) have the hospital services and non-ministry alternatives located in these regions. Closing the gate, especially in these regions, would create an access burden, rather than a workforce burden. This study cannot study that, as it studies supply and not the utilization. It can state that the equity consequences of the referral policy depend on the distribution found in this study, and that sequencing is important. Closing the gate before the distribution is corrected would be most detrimental in the regions that are least able to deal with the consequences. When considering the Vision 2030 objectives, the findings do not provide unequivocal support or criticism.

Health objectives focus on universal health coverage, shift in health service activity from hospitals to primary care, larger and more Saudi workforce, and improved health service access. The trends described here are consistent with progress on several of these objectives, although a descriptive series cannot show that the programme caused them. The number and density of family physicians increased by 62.8% and 53.0%, respectively, over the period of nine years. The growth was fastest in the regions that had least (7, 9, 12). For the access objective the picture is more complex, because access is a function of distribution, and the distribution has actually become less even. It is important to describe this unevenness because it is not the failure mode that equity-oriented programs aim to address. The regions that are lagging behind are not remote and underserved regions. They are large urban regions and they are lagging behind in a ministry employed workforce, while there are hospitals, universities, and private institutions. The most reasonable interpretation is that Vision 2030 has partially achieved its redistributive goals, which has resulted in a secondary concern that the program monitoring framework is unable to detect. This concern is that while urban populations continue to grow, there is a primary care viability issue in urban centers. Adding a distributional measure to the framework is cost effective.

Reporting the Gini coefficient of regional family physician density, compared to the national ratio, provides the opportunity to formally assess the division of resources equitably. The division of resources from an equity perspective would mirror the evaluation of a rising bar of the growth objective.

At present, there is a disparity where the gap in the growth objective is analyzed and evaluated, but the division of resources is not evaluated from an equity perspective. This is because resources are not formally assessed to see if they are equitably distributed.

## 5 In regard to the perspective of resource growth, the division of resources and the equity of distribution are also analyzed

The results also indicate that there is a dimension that has methodological potential. Administrative boundaries are containers, and an index determined over containers indicates inequality at the level of the container. A province can register an adequate ratio, if one well-staffed urban cluster of centers offsets a large, under-served rural area. Nothing in the Gini coefficient reported here would recognize that inequality. The restriction is eliminated by Geographic Information Systems (GIS), which rely on the coordinates of facilities and population grids, rather than sums of administratively delineated units. The two-step floating catchment area method and its distance-decay variants produce an accessibility score for each populated location by weighing the supply at each facility with the demand and the impedance of travel (41). Two things are evident that regional ratio conceals: under-served areas in seemingly well-served regions, and that the served areas lie in facilities that cross an administrative boundary. Similar studies have been conducted on health facilities in the Kingdom, including a governorate-level investigation of dental services in Jazan that indicated the great majority of the clinics were concentrated within twenty kilometers of city centers, as well as a national study of primary health care clinics in relation to the health workforce (18, 42). Combining the count of family physicians with the facility coordinates, the road network and a gridded population would be able to address the questions that this design is not able to answer.

How much of the between-region inequality is reproduced within regions? This question helps us identify which populations are beyond a given travel-time threshold for family physicians. How much additional coverage do new posts provide? The last question transforms an equity diagnosis into a site planning instrument. In the context of this study, it is the most challenging question. The Ministry has the site coordinates necessary to answer this last question.

Looking at the changes holistically, the health reform period coincided with substantial quantitative growth in the MOH family medicine workforce, and that growth was concentrated in the regions that had the least to begin with. Regional inequality in the distribution of family medicine needs more attention. Using the indicators provided, the last regional inequality was actually higher than the first. Distributing the gains of regional growth into more equitable forms of regional growth is the challenge of the next phase. This study provides a baseline with which future improvements can be measured. The consolidation phase can be built upon a strong foundation of sustaining growth and targeted retention incentives in the high-growth periphery coupled with the slow-growing urban core, alongside a concerted effort to track regional inequalities.

### 5.1 Implications

The findings have clear implications for workforce planning. Since the binding constraint is distributional rather than aggregate, the subsequent phase of the health reform can focus on equity while continuing to promote growth. This would mean moving beyond the large urban centers for family medicine residency training. It would mean offering region-specific retention incentives to the slower growing urban clusters and protecting the gains made in rural areas. Planners would be able to monitor regional density regularly and, using the Gini or Theil indices, identify and address regional inequities and evaluate the “a family physician for every family” initiative based on equity. From the analysis in Sections 3.7 and 3.8, three additional points can be made. The equity gap in the metropolitan units pertains to ministry-employed staff, so the first response needs to be a cross-sectional workforce audit in those cities and not an immediate recruitment drive. Equity regional planning goals will tend to overlook the most important inequitable areas. This means that, along with regional goal planning, subregional planning needs to be done.

### 5.2 Strengths and limitations

The strengths and weaknesses of this paper are explained below. The longitudinal, balanced-panel design for the full 2014–2022 period captures the family medicine workforce and the entirety of the workforce spanning the three phases of the reform. This captures the family medicine workforce rather than a single cross-sectional component of the workforce. Additionally, using the three indices of inequality (Gini, Theil, and CV) along with the bootstrap confidence intervals provides a strong and comprehensive approach to the selection of the indices. Finally, the clustering analysis provides an interpretable regional typology of the trajectories complemented by the aggregate inequality measures.

This study has three main strengths. This is the first study to analyze family medicine in the post-reform period. This allows for the opportunity to compare the post-reform distribution with a legitimate pre-reform baseline. This study uses three inequality indices while also using bootstrap confidence intervals. Lastly, a single cohesive source of data is used to eliminate confounding variables caused by multiple sources, something that affects most similar studies in family medicine. The limitations of this study are noted below.

Limitations are fourfold. First, the post-reform phase is set to two years (2021–2022) instead of four, as the Statistical Yearbooks for 2023 and 2024 have not yet been released in a comparable format. The analysis of the full 2021–2024 data will be possible only after the yearbooks are released. Second, the analysis is done on the basis of data from the Ministry of Health. Therefore, data on the private sector and military medical services is not included. Third, the 2021 reclassification as part of the migration to regional health clusters caused a gap in the workforce data, for which data publication is not able to provide a reason. 2021 is therefore treated as an administrative artifact, and the artifact-adjusted estimates are given precedence over the full series. Fourth, the design is descriptive and uncontrolled, with no comparison group and no counterfactual. It shows how the distribution changed alongside the reform, but it cannot establish that the reform caused those changes, and the language used throughout the paper is meant to reflect that. Therefore, the redistributive policy in favor of the periphery is attributed to some of the Saudization policies, to the expansion of training, to the effects of some of the redistribution policies, to the workforce incentives. The fourteen-unit administrative classification of the MOH is different from the thirteen-region civil-administrative classification used in some other Saudi datasets. Any comparison and the definitions of the units are necessary for any direct comparison with non-MOH workforce data.

The data source in question is worth mentioning because it is central to how we interpret the urban data. The Statistical Yearbooks only include Ministry of Health facilities. The delivery of primary and ambulatory care by the private sector, the security forces and National Guard, Ministry of Defense, and university medical cities is completely beyond the scope of the data source. This is not a geographically neutral omission. Non-Ministry provision is heavily skewed towards Riyadh, Jeddah, Makkah, and the Eastern Province, which coincidentally are the units that show stagnation or decline in this series. A family physician who moves from a Ministry of Health center in Jeddah to a private polyclinic two kilometers away leaves this dataset without leaving the city, and the shift to cluster-based governance, corporatization, and increased private participation, during the study period, propensity for this type of movement, increased. Therefore, the stagnation observed in the metropolitan units should be interpreted as a Ministry of Health-related stagnation of family physicians and should not be understood as a family physician shortage of the cities. The stagnation in family physicians would only become evident if workforce data from all sectors was integrated, which, unfortunately, does not exist on a regional basis. For the sake of accuracy, all statements of urban stagnation in this paper was written with the proper understanding that stagnation in family physicians does not signify a shortage. Therefore, readers should not draw conclusions on total local supply based on any statements made in this paper.

## 6 Conclusion

This study describes changes in the regional distribution and equity of the MOH-employed family medicine workforce in Saudi Arabia over the nine years of available administrative data that span the Vision 2030 reform. During this period, family medicine workforce density has increased significantly and in a positive direction. There were increases in family medicine workforce density in all regions, accompanied by widening inequities in all three measures (D1, D2, and D3) in 2022 compared to 2014. The increase was concentrated in the last two years of the data. The endpoint comparison is not affected by the 2021 reclassification, but the monotonic trend is only marginally significant when 2021 is included (*p* = 0.048) and not significant when it is excluded (*p* = 0.108), so the widening should be regarded as suggestive rather than robust. Within the MOH workforce, these patterns differ from the urban bias usually observed internationally, as increases in peripheral regions have been accompanied by stagnation or declines in the major urban centres. Because non-MOH providers are concentrated in those cities and are not captured by the data, this finding describes MOH provision and should not be read as a reversal of urban bias in total family physician supply.

The policy implication is that the next phase of the reform should focus on achieving equity, instead of looking for more aggregate growth. Four measures are proposed. These are based on the findings and are not general recommendations. First, retention incentives should target five units: Jeddah, Makkah, Al-Ahsa, Hail and Hafar Al-Batin. These incentives should be post differentials and not national uplifts. A national uplift moves every region and does not address the gap. Second, in the next planning cycle, the capacity of the Northern Borders, Bisha and the Southern units should be maintained instead of being rebalanced toward the center. These units achieved the gains, and the gains are recent. Third, special attention should be paid to the monitoring and reconciliation of Riyadh. The post-reform density of 0.99 per 10,000 of the Riyadh region is most important. Until the 2021 reassessment is reconciled against the cluster payroll, it cannot be determined how much of this change is real. Maintaining special attention and focus on reconciliation of this region is important. Fourth, as part of routine reporting, the Gini coefficient of regional family physician density should be published as an index along with the national ratio so that it can serve as a threshold to warrant distribution monitoring. The index used in this analysis is low cost, and can easily be computed and reproduced from the Ministry published data.

For the next expansion round, spatial accessibility analysis at the sub-regional scale should be preferred for situating new facilities and posts. This should be done prior to the next expansion round instead of after. Followed by the measures above, the consolidation phase would be able to advance toward realizing the Vision 2030's goal of increasing the health care workforce while balancing the demand for family medicine services across the Kingdom.

## Data Availability

The original contributions presented in the study are included in the article/Supplementary Material, further inquiries can be directed to the corresponding author.
